# The complete mitochondrial genome of *Paraqianlabeo lineatus* (Cyprinidae: Labeoninae)

**DOI:** 10.1080/23802359.2021.1907799

**Published:** 2021-04-08

**Authors:** Xue Wang, Sheng Zeng

**Affiliations:** a Guizhou Fisheries Research Institute, Guiyang, P. R. China; b Guizhou Academy of Agricultural Sciences, Guiyang, P. R. China

**Keywords:** *Paraqianlabeo lineatus*, mitochondrion, Labeoninae, phylogeny

## Abstract

*Paraqianlabeo lineatus* is a small-sized fish which is endemic to Guizhou province, China. The complete mitochondrial genome of *P. lineatus* is 16,598 bp in total length, with 37 genes, including 13 PCGs, 22 tRNA genes, two rRNA genes (16S and 12S) and a non-coding region (D-loop). The positions and sequences of genes were consistent with congeners of Labeoninae. The nucleotide composition of the mitogenome was A (31.5%), T (26.7%), G (15.9%), C (25.8%) and was slightly A + T biased. Phylogenetic analysis conducted using Bayesian Inference method showed that *P. lineatus* clustered with *Pseudogyrincheilus procheilus* within the subfamily Labeoninae. The results may provide helpful data for further studies of the evolutionary history of Labeoninae.

*Paraqianlabeo lineatus* is a small-sized fish which is endemic to drainages of Yangtze river, Chishui river and Wu-Jiang river, Guizhou province, China (Zhao et al. 2014). This monotypic species is usually found in upper hill streams with rocks. There are few ecological and molecular data about *P. lineatus* (Li et al. 2016; Zhao 2016). Here, we provide the first description of the complete mitochondrial genome of *P. lineatus*. The sequence was obtained from a specimen caught in Mengxi river, Shizhi village, Zhengan county (E 107°17′37″, N 28°17′17″), Guizhou province, a tributary flowing into Wu-Jiang river. Total genomic DNA was extracted from the pelvic fin preserved in 95% alcohol using the Qiagen QIAamp tissue kit following the manufacturer’s protocol. The complete mitochondrial genome was sequenced by next-generation sequencing methods and the annotated sequence was archived at Genebank (accession number MW039085). The specimen (GZ-20170217, 7.7 cm in standard length, 5.0 g in weight) was deposited in the collection of fisheries research institution, Guizhou Academy of Agricultural Sciences.

Structure of the mitogenome of *P. lineatus* was consistent with those of other fishes (Zheng and Yang 2016; Tan et al. 2019; Pan et al. 2020), consisting 13 protein-coding genes, 2 ribosomal RNA (rRNA) genes, 22 transfer RNA (tRNA) genes, and a non-coding regions. Most of the coding regions are encoded on the H-strand except for *ND6,* and there are eight tRNA genes (tRNA^Gln^, tRNA^Ala^, tRNA^Asn^, tRNA^Cys^, tRNA^Tyr^, tRNA^Ser^, tRNA^Glu^ and tRNA^Pro^) encoded on H-strand. The base composition of protein-coding genes showed a weak anti-G bias (15.3%) whereas the anti-G bias was strongly observed in the third codon position (6.7%), overall base composition of A + T content (58.2%) is nearly the same with coding region genes (58.8%). Almost all of the 13 protein-coding genes started with the typical start codon ATG but *COI,* which started with GTG. For the stop codon, six of them share the complete stop codon, while seven shared the incomplete stop codon with a terminal T or TA. The D-Loop was located between tRNA^Pho^ and tRNA^Phe^ with 936 bp, the base composition reflected a strong A + T-rich (65.7%), richer than the overall average content (58.2%).

We chose Bayesian Inference method to infer evolutionary relationships based on complete mitogenome between *P. lineatus* and 15 other sequences downloaded from Genebank*, Acrossocheilus yunnanensis* (Cyprinidae: Barbinae) was chosen as outgroup here. The phylogenetic tree showed that *P. lineatus* clustered with *Pseudogyrincheilus procheilus* with high Posterior Probability (Figure 1), consistent with previous studies based on short mitochondrial DNA sequences (Zhao 2016; Zheng et al. 2016). this may tell us that genus *Paraqianlabeo* and *Pseudogyrincheilus* have more closely evolutionary relationship, and the results would provide references for further study of subfamily Labeoninae.

**Figure 1.**
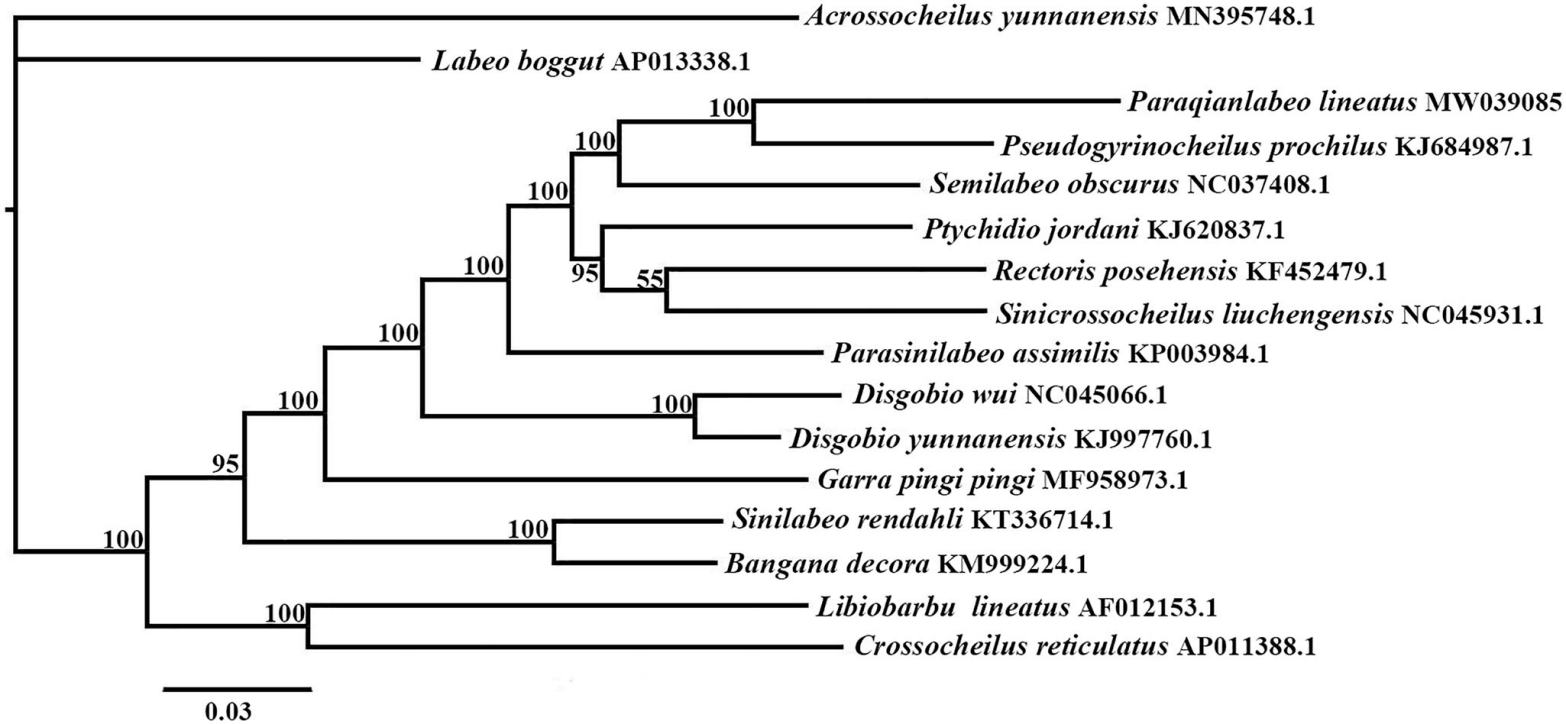
Phylogenetic relationships between *P. lineatus* and 15 other members of Labeoninae based on complete mitochondrial genome sequences. *Acrossocheilus yunnanensis* was used as an outgroup. Numbers on the nodes are Bayesian posterior probability values.

## Data Availability

The genome sequence data that support the findings of this study are openly available in GenBank of NCBI at (https://www.ncbi.nlm.nih.gov/) under the accession no. MW039085. The associated BioProject, Bio-Sample and SRA numbers are PRJNA694796, SAMN17574689 and SRR13664247 respectively.
